# Mothers’ experiences of caring for a child with acute lymphoblastic leukemia in the West Bank, Palestine: A qualitative study

**DOI:** 10.1371/journal.pone.0362098

**Published:** 2026-11-04

**Authors:** Baraa Ghalib Yassin Taqatqa, Kawther Elissa, Amani Salim, Salam Khatib

**Affiliations:** 1 Department of Nursing, Faculty of Health Professions, Al-Quds University, Jerusalem, Palestine; 2 Faculty of Midwifery, Bethlehem University, Bethlehem, Palestine; Teda Health Science College, ETHIOPIA

## Abstract

**Background:**

Acute lymphoblastic leukaemia (ALL) is the most common childhood cancer globally and requires prolonged, intensive treatment that places substantial demands on families. Mothers often assume primary caregiving roles; however, their experiences in conflict-affected settings remain underexplored.

**Objective:**

This study aimed to explore the experiences of Palestinian mothers caring for a child diagnosed with ALL in the West Bank.

**Methods:**

A qualitative descriptive study included fifteen mothers of children diagnosed with ALL and receiving treatment at Beit Jala Governmental Hospital, who were recruited using purposive sampling. Data were collected through semi-structured interviews, transcribed verbatim and analyzed using qualitative content analysis following the approach of Graneheim and Lundman (2004).

**Results:**

The study identified five major themes and eighteen sub-themes reflecting mothers’ experiences: emotional issues (shock and disbelief, negative emotions, denial and rejection); hindrances due to political situations (limited health care access, feeling isolated due to closures); impact of the child’s illness (caregiver burden vs. protective bonding, financial strain, loss of previous life, sibling neglect, shift in the role of the mother, marital life impact, challenges in balancing home routine); resilience and endurance (leveraging social support networks, fatalism and religious faith as a framework, cultivating optimism and positive outlook); and mothers’ needs to enhance resilience (emotional and psychological support, educational and informational needs, financial needs).

**Conclusion:**

Mothers caring for children with ALL in the West Bank face profound emotional, social, and structural challenges shaped by both illness-related and political contextual factors. Despite these adversities, mothers demonstrate resilience through social support networks, religious faith, and optimism. Family-centered and context-sensitive interventions that address emotional, educational, and financial needs are essential for strengthening maternal resilience and improving care outcomes.

## Introduction

Acute lymphoblastic leukemia (ALL) is the most common malignancy in childhood, accounting for approximately 25–30% of all pediatric cancers worldwide [1]. Advances in early diagnosis, risk stratification, and treatment protocols have led to substantial improvements in outcomes, with five-year survival rates now exceeding 85% in many settings [2]. As survival has improved, childhood ALL has increasingly become a chronic condition requiring prolonged and intensive treatment, placing sustained emotional, social, and practical demands on families and caregivers.

Parents, particularly mothers, play a central role in the care of children with ALL. Previous studies consistently show that mothers experience profound psychological distress following diagnosis, including shock, fear, anxiety, uncertainty, and feelings of helplessness [3,4]. These emotional responses may persist throughout treatment and beyond, driven by fear of relapse and concerns about long-term physical and psychosocial consequences for the child [5,6]. In addition to emotional strain, caregiving responsibilities often disrupt family routines, employment, social relationships, and marital dynamics, and may negatively affect relationships with siblings [7,8].

Caregiving experiences are strongly shaped by the broader social, economic, and healthcare context. In low-resource and conflict-affected settings, families of children with cancer face compounded challenges, including delayed diagnosis, limited access to specialized oncology services, financial hardship, and interruptions in treatment [9]. In Palestine, access to healthcare is further constrained by political and structural factors such as military checkpoints, permit systems, road closures, and fragmentation of health services. These barriers have been shown to delay cancer diagnosis and treatment, disrupt continuity of care, and exacerbate psychological distress among patients and their families [10,11].

Despite growing international literature on mothers’ experiences of childhood cancer, there remains a significant gap in qualitative research focusing on caregivers in Palestine and other conflict-affected contexts. Most existing studies originate from high-income or politically stable countries and may not adequately capture the intersecting medical, political, and socioeconomic stressors faced by Palestinian mothers caring for a child with ALL. Addressing this gap is essential to inform culturally sensitive, family-centered pediatric oncology care. Therefore, this study aimed to explore the experiences of Palestinian mothers caring for a child diagnosed with acute ALL in the West Bank, focusing on emotional, social, practical, and contextual challenges, as well as coping strategies and sources of support.

## Methods

### Study design

The study employed a qualitative descriptive design, utilizing individual interviews and analyzed through qualitative content analysis. Qualitative research aims to understand individuals’ inner experiences and perspectives. Since personal experiences shape how individuals interpret their experiences, researchers can uncover the meaning of phenomena by examining these experiences from the participants’ point of view [12]. This approach is particularly suitable for studies aiming to describe and interpret a phenomenon, as was the case in the present study [13].

### Study setting

The study was conducted at the Huda Al-Masri Pediatric Cancer Department, a dedicated pediatric oncology unit within Beit Jala Governmental Hospital (Al-Hussein Hospital), Bethlehem Governorate, West Bank, Palestine. The hospital was purposively selected as the study setting because it is the only governmental pediatric oncology unit serving children with cancer in the south, north, and middle regions of the West Bank, making it a key setting for accessing children with leukemia receiving treatment in these regions. The setting was identified based on its specialized pediatric oncology services and the availability of eligible patients receiving ongoing treatment. This public hospital, operated by the Palestinian Ministry of Health, has a reported capacity of 131 beds. The department provides inpatient care, diagnostic services, chemotherapy, and psychosocial support for children with leukemia and other malignancies, including isolation facilities with 14 beds (hospital administrative records; recently equipped with 14 electric beds [14].

### Participants and recruitment

Purposive sampling was used to recruit mothers of children diagnosed with acute ALL who were receiving treatment in the pediatric hematology department. Eligible participants were mothers residing in the West Bank whose child had been diagnosed with ALL for at least four months and who agreed to participate. Mothers were excluded if their child had been diagnosed with ALL less than four months previously or if they declined to participate. The four-month criterion was applied to ensure that mothers had sufficient experience of their child’s illness and treatment to provide detailed accounts of their caregiving and emotional experiences. No eligible mother declined participation, and all 15 mothers who were approached and met the eligibility criteria participated in the study.

Mothers were not excluded based on receiving psychological, emotional, or other supportive interventions related to their child’s illness, and palliative care was not specified as an exclusion criterion. Treatment modality was not used as an eligibility or exclusion criterion, although differences in treatment type and intensity were considered when interpreting the findings because they may influence caregiving demands and emotional experiences.

Approval to recruit participants was obtained from the Palestinian Ministry of Health on 29 January 2024. Potentially eligible participants were identified with assistance from the head nurse and a senior nurse in the pediatric hematology department. Eligible mothers were informed about the study and invited to participate voluntarily. Interviews were conducted at a time and location convenient for each participant. Purposive sampling sought variation in relevant sociodemographic and clinical characteristics, including maternal age, educational level, place of residence, and the child’s age and treatment duration, to capture diverse caregiving experiences and enhance the transferability of the findings.

Recruitment and data collection proceeded iteratively, with emerging findings reviewed throughout the process. Data saturation was reached after 15 interviews, when the final interviews generated no substantively new codes, categories, or themes and confirmed the patterns identified in earlier interviews regarding experiences of Palestinian mothers caring for a child diagnosed with ALL in the West Bank. The decision that saturation had been reached was made collaboratively following repeated discussions of emerging findings during the analytic process among research team.

### Data collection

Data were collected through face-to-face, semi-structured interviews conducted by the first author, who holds a Master’s degree in Maternal and Child Health Nursing and works as a researcher. The research team had prior experience and training in qualitative research and qualitative interviewing. The interviewer was female and had no prior professional relationship with the participating mothers before recruitment. Before each interview, participants were informed about the interviewer’s professional background, the purpose of the study, and her role as a researcher. The interviewer did not provide clinical care to the participants during the study. Participants were formally enrolled in the study between 10 February 2024 and 20 May 2024. All Interviews were conducted individually in a quiet and private setting within the hospital, according to participants’ preferences. No family members, healthcare professionals, or other individuals were present during the interviews. All interviews were audio-recorded with participants’ consent. Each participant was interviewed once, and no repeat interviews were conducted.

The interviews were conducted in Arabic, the native language of the participants and the research team. They were subsequently transcribed verbatim and translated into English for analysis. To ensure the credibility and conceptual accuracy of the translation, the transcripts were independently reviewed by multiple bilingual members of the research team, with discrepancies discussed and resolved through consensus. Reflexive notes were maintained throughout data collection and analysis to document the interviewer’s assumptions, observations, and potential influence on the interview process and interpretation.

The average interview duration was about 40 minutes. An interview guide was used, and the following main questions were asked of the mothers: “Can you tell me about the family situation before and after you learned that your child had ALL?”, “How did you feel and react when you knew that your child had ALL?”, “What challenges do you face in helping your child manage his illness? (financial, social, medical)”, “Can you describe your experiences in accessing healthcare services for leukemia patients in the West Bank?” and “Could you describe what factors helped you cope during your child’s treatment and hospital stay?” Follow-up questions were used to obtain more detailed information, such as “What do you mean by that?” “How do you feel about that?” and “Can you give me an example?” The interview guide was reviewed by qualitative research experts and pretested through two preliminary interviews to confirm its credibility and consistency.

Throughout the data collection process, sensitivity to participants’ emotional states was prioritized. Interviews were conducted flexibly, allowing breaks or rescheduling when needed to ensure a respectful and supportive environment. Field notes were recorded immediately after interviews to document contextual observations, participants’ emotional expressions, and reflections relevant to the interpretation of the data.

### Data analysis

Qualitative content analysis was conducted using the approach described by Graneheim and Lundman [15]. This approach was selected because it provides a systematic process for identifying and interpreting patterns within textual data while allowing consideration of both manifest and latent content.

All interviews were transcribed verbatim and checked against the audio recordings for accuracy. The transcripts were read repeatedly by the researchers to facilitate immersion in the data and to develop an overall understanding of the mothers’ experiences. Meaning units relevant to the study aim were identified and condensed while retaining their essential meaning. The condensed meaning units were then assigned descriptive codes. Codes with conceptual similarities were compared, discussed, and progressively grouped into subthemes and broader themes through an iterative process of abstraction. An example of the analytical process from meaning unit to condensed meaning unit, code, subtheme, and theme is presented in Table 1.

**Table 1:** Examples from the Analytical Process.

Meaning Unit	Condensed Meaning Unit	Code	Sub-theme	Theme
The service here is worse due to the roads, the occupation, and the many closures. It is very difficult for me to get from Hebron to Bethlehem.	Health services are compromised by occupation-related restrictions, making intercity travel difficult.	Difficulty accessing health services due to mobility restrictions	Limited healthcare access	Hindrances due to the political situation
I was greatly affected. My role as a mother in the home was affected. I became a very negligent mother in my role at home, negligent with my children, my husband, and my housework.	Maternal caregiving and household responsibilities were negatively affected by the child’s illness.	Disruption of maternal role and family responsibilities	Family role disruption/caregiver strain	Impact of the child’s illness
My son’s illness was a test for my family and me. God gave me a lot. He gave me a family, a home, children, and a job. He wanted to test how I am...and thank God.	Illness is perceived as a divine test, accompanied by acceptance and gratitude.	Illness interpreted as a divine test with acceptance	Fatalism and religious coping	Resilience and endurance

To enhance analytical credibility and reduce the influence of individual interpretation, the transcripts were initially analysed independently by the researchers. The researchers subsequently compared their coding and interpretations and discussed similarities and differences in the identified meaning units, codes, subthemes, and themes. Any disagreements were resolved through discussion and consensus, with reference to the original transcripts and participants’ accounts when necessary. The research team repeatedly returned to the raw data throughout the abstraction process to ensure that the developing interpretations remained grounded in participants’ narratives. The final themes and subthemes were reviewed collectively by the research team for internal coherence, distinctiveness, and consistency with the study objectives.

No qualitative data analysis software was used. Data were organized and analysed manually using the transcripts and structured analytical tables. This process supported an iterative movement between the original data and the emerging codes, subthemes, and themes throughout the analysis.

### Trustworthiness

Rigor in this qualitative study was ensured by addressing the criteria of credibility, dependability, confirmability, and transferability, as described by Graneheim and Lundman [15]. Credibility was enhanced through purposive sampling aimed at capturing variation in relevant sociodemographic and clinical characteristics, iterative engagement with the data, and repeated comparison of emerging codes, subthemes, and themes with the original transcripts. The researchers remained engaged with the data throughout the analytical process and repeatedly returned to participants’ narratives to ensure that interpretations were grounded in the data. A back-translation process was used to enhance the linguistic accuracy and cultural appropriateness of interview data translated from Arabic into English.

Dependability was supported through the use of a structured interview guide and systematic documentation of the research process, including recruitment, data collection, coding, theme development, and analytical decisions. The researchers independently reviewed the transcripts before comparing their interpretations. Differences in coding or interpretation were discussed among the research team and resolved through consensus, with reference to the original transcripts. This iterative process supported consistency and transparency throughout the analysis.

Confirmability was strengthened by explicitly acknowledging the potential influence of researcher bias arising from the researchers’ professional backgrounds, prior clinical experiences, and assumptions. The research team included researchers with backgrounds in nursing, public health, mental health and maternal and child health. While this expertise provided valuable contextual understanding, the researchers recognized that their professional perspectives could influence data collection and interpretation. Reflexive notes were therefore maintained throughout data collection and analysis, and researchers critically examined their assumptions and emerging interpretations. During analysis, researchers independently reviewed the transcripts before collaboratively comparing codes, subthemes, and themes.

Differences in interpretation were discussed and resolved through consensus, with repeated reference to the original transcripts and participants’ accounts. The research team also critically considered whether emerging interpretations were adequately supported by the data or reflected researchers’ preconceptions. These procedures were used to minimize the potential influence of individual researcher perspectives and ensure that the findings remained grounded in participants’ narratives.

Transferability was supported by providing detailed descriptions of the study context, participant characteristics, recruitment procedures, and healthcare setting. Purposive sampling sought variation in participants’ sociodemographic and clinical backgrounds to capture diverse caregiving experiences. These contextual details enable readers to assess the potential applicability of the findings to mothers caring for children with cancer in comparable healthcare and conflict-affected settings.

Transcripts and preliminary findings were not returned to participants for member checking because of the sensitive nature of the topic and the practical constraints associated with participants’ ongoing caregiving and treatment responsibilities. Instead, credibility and confirmability were strengthened through independent review of the transcripts, comparison and consensus among the research team, repeated reference to the original transcripts and participants’ accounts, and reflexive documentation. This approach ensured that the interpretation remained closely grounded in participants’ narratives while acknowledging and addressing the potential influence of researcher perspectives.

The study was reported in accordance with the Consolidated Criteria for Reporting Qualitative Research (COREQ) guidelines.

### Ethical considerations

Ethical approval for the study was obtained from the Research Ethics Committee at Al-Quds University (Ref: 346/REC/2023). Additional permission to conduct the study was granted by the Palestinian Ministry of Health and the hospital administration.

All participants were provided with verbal and written information explaining the purpose, nature, and aims of the study, as well as their right to refuse participation, withdraw at any time, or skip any question without consequences. Written informed consent was obtained from all participants before data collection, including consent to audio-record the interviews. Confidentiality was strictly maintained throughout the study. Participants’ identities were anonymized using identification numbers, and all data were stored securely and accessed only by the research team.

## Results

### Participants

A total of 15 mothers of children diagnosed with leukaemia participated in this study (see Table 2). The mothers’ ages ranged from 20 to 49 years, with most aged 30–39 years (46.7%). All participants were married and Muslim (100%). Educationally, the majority had completed high school (53.3%) or held a bachelor’s degree (33.3%), and most were homemakers (80%). Participants primarily resided in villages (80%), with monthly family incomes of 1500–2000 ILS, which equals ($405–$541 USD) (46.7%) and family sizes of 4–6 members (40%). All mothers reported having governmental health insurance (100%).

**Table 2:** Demographic and occupational characteristics of mothers and children (n = 15).

Variables	Category	Number (n)	Percentage (%)
**Mother’s Age (years)**	<19	0	0
	20–29	3	20
	30–39	7	46.67
	40–49	5	33.33
	>50	0	0
**Marital Status**	Married	15	100
	Divorced	0	0
	Widowed	0	0
	Separated	0	0
**Mother’s Education Level**	Middle school	2	13.33
	High school	8	53.33
	Bachelor’s degree	5	33.33
**Mother’s Occupation**	Homemaker	12	80
	Employed	3	20
**Place of Residence**	City	2	13.33
	Village	12	80
	Refugee camp	1	6.67
**Religion**	Muslim	15	100
	Christian	0	0
**Monthly Income (ILS)**	<1500	1	6.67
	1500–2000	7	46.67
	2000–3000	5	33.33
	>3000	2	13.33
**Family Size**	1–3 members	2	13.33
	4–6 members	6	40
	7–9 members	6	40
	>10 members	1	6.67
**Type of Health Insurance**	Governmental	15	100
**Child’s Age (years)**	2.5–3.5	5	33.33
	4	3	20
	5	3	20
	6	2	13.33
	8	1	6.67
	12	1	6.67
**Child’s Gender**	Male	7	46.67
	Female	8	53.33
**Duration of ALL Diagnosis**	<1 year	6	40
	2–3 years	7	46.67
	>3 years	2	13.33
**Place of Treatment**	Beit Jala Governmental Hospital – Huda Al-Masri Ward	15	100

The children’s ages ranged from 2.5 to 12 years, with a nearly equal distribution of males (46.7%) and females (53.3%). The duration of leukaemia diagnosis varied, with most children diagnosed within 2–3 years (46.7%). All children received treatment at Beit Jala Governmental Hospital – Huda AlMasri Ward.

### Themes

The study analysis generated five major themes and eighteen sub-themes reflecting mothers’ experiences while caring for a child with a serious illness in the West Bank. These themes include (1) emotional issues (shock and disbelief, negative emotions, denial, and rejection); (2) hindrances due to the political situation (limited healthcare access and feeling isolated due to closures); (3) the impact of the child’s illness (caregiver burden versus protective bonding, financial strain, loss of previous life, sibling neglect, shift in the role of the mother, marital life impact (positive and negative), and challenges in balancing home routine); (4) resilience and endurance (leveraging social support networks, fatalism and religious faith as a framework, and cultivating optimism and a positive outlook); and (5) mothers’ needs to enhance resilience (emotional and psychological support, educational and informational needs, and financial needs) as Table 3. Together, these themes illustrate the complex emotional, social, and contextual challenges mothers faced, as well as the strategies they used to cope and adapt.

**Table 3:** Themes and Sub-themes of the Experiences of Mothers Having a Child with Leukemia in the West Bank, Palestine.

Theme	Sub-themes
**1. Emotional issues**	1.1 Shock and disbelief 1.2 Negative emotions 1.3 Denial and rejection
**2. Hindrances due to political situations**	2.1 Limited health care access 2.2 Feeling isolated due to closures
**3. Impact of the child’s illness**	3.1 Caregiver burden vs. protective bonding 3.2 Financial strain 3.3 Loss of previous life 3.4 Sibling neglect 3.5 Shift in the role of the mother 3.6 Marital life impact (positive and negative) 3.7 Challenges in balancing home routine
**4. Resilience and Endurance**	4.1 Leveraging social support networks 4.2 Fatalism and religious faith as a framework 4.3 Cultivating optimism and positive outlook
**5. Mothers’ needs to enhance resilience**	5.1 Emotional and psychological support 5.2 Educational and informational needs 5.3 Financial needs

The interconnected nature of these experiences is illustrated in Fig 1, which presents a visual model of experiences of mothers caring for a child with ALL in the West Bank.

**Fig 1:**
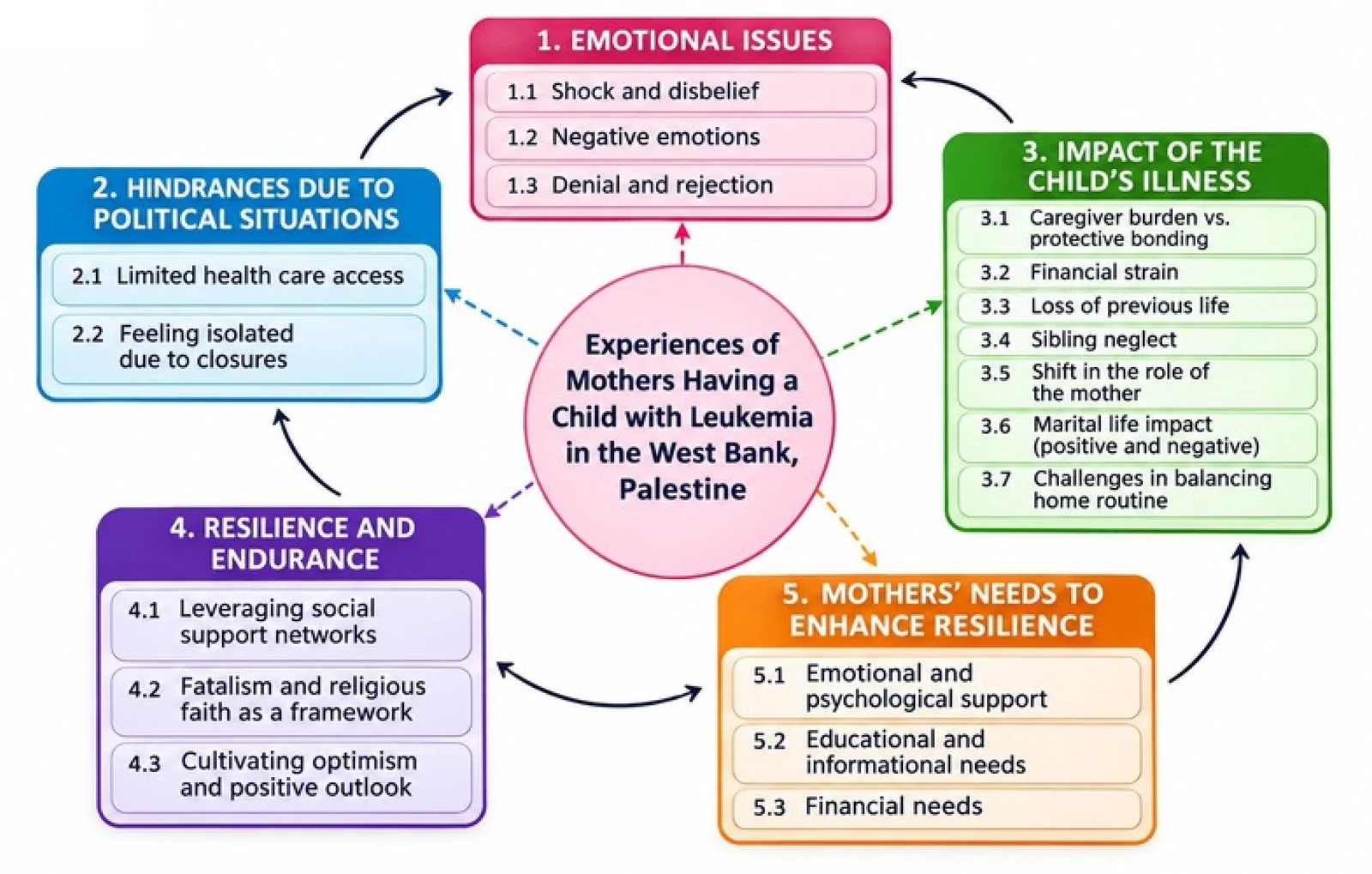
Visual model of the experiences of mothers caring for a child with ALL in the West Bank.

Hindrances due to political situation, including limited healthcare access and feelings of isolation due to closures, contribute to emotional issues, such as shock, negative emotions, and denial. The impact of the child’s illness also creates substantial practical and social challenges, including disruption of previous life, sibling neglect, changes in the mother’s role, effects on marital life, and difficulties balancing household routines, which may further intensify emotional distress. In response, mothers draw on resilience and endurance through social support, religious faith, and optimism. Their capacity to cope is closely linked to access to emotional and psychological, educational and informational, and financial support. Overall, the model depicts a dynamic process in which political and illness-related challenges shape mothers’ emotional and practical experiences, while resilience strategies and supportive resources facilitate coping and adaptation.

#### Emotional issues.

The first theme identified from the data analysis was emotional issues, reflecting the profound psychological impact of a child’s leukaemia diagnosis on mothers. Participants described experiencing intense emotional distress upon learning about their child’s illness, often accompanied by feelings of uncertainty and fear about the future. The diagnosis represented a sudden and life-altering event that challenged mothers’ emotional stability and coping capacity. These emotional reactions highlighted the difficulty of processing such unexpected news and the burden associated with anticipating the demanding treatment journey ahead. This theme comprised three subthemes: shock and disbelief, negative emotions, and denial and rejection.

**Shock and disbelief.** Many mothers described their initial reaction to the diagnosis as one of shock and disbelief. The unexpected nature of the news made it difficult for them to immediately comprehend or accept the reality of their child’s illness. Several participants reported feeling confused, emotionally overwhelmed, and temporarily unable to process the information provided by healthcare professionals.

*“I couldn’t believe or understand that my daughter had this disease. I was just confused when the doctor told us; it felt impossible.”* (Mother of a 3.5-year-old female)

*“It was the shock of my life; I never expected this and refused to believe my son was sick, thinking the tests were wrong.”* (Mother of a 4.5-year-old male)

*“Imagine your daughter suddenly diagnosed with a serious illness, enduring pain, medication, and chemo. It’s the shock.”* (Mother of a 3.5-year-old female)

*“My first reaction was one of disbelief and confusion about what was happening. I feel like I was slapped by someone.”* (Mother of a 3.5-year-old female)

**Negative emotions.** Following the initial shock, mothers described experiencing a range of negative emotions, including fear, anxiety, helplessness, and emotional distress. These feelings were often intensified by uncertainty regarding the child’s prognosis and the complexity of treatment procedures. Participants also reported concerns about their child’s future and their ability to manage caregiving responsibilities during the prolonged treatment period. In many cases, these emotional responses had a significant impact on mothers’ psychological well-being.

*“The entire period of illness was filled with fear, stress, and anxiety.”* (Mother of a 6-year-old male)

*“I felt lost and confused about the illness, which heightened my anxiety and discomfort.”*(Mother of a 3-year-old female)

*“I couldn’t handle her; only I could feed and calm her. Sometimes I’d sit and cry, feeling helpless.”*(Mother of a 3.5-year-old female)

*“I have deep concerns about my daughter’s future, especially regarding potential relapses and whether she will fully recover or face challenges in life.”* (Mother of a 3.5-year-old female)

*“I faced intense emotional strain, to the point where I asked my mother to take my child for treatment; I felt an overwhelming urge to escape from everything.”*(Mother of a 4-year-old male)

*“I’m afraid of losing my child, so I always stay with him.” (*Mother of a 6-year-old male)

**Denial and rejection.** Some mothers initially responded to the diagnosis with denial and rejection, reflecting the psychological difficulty of accepting such a distressing reality. Participants described questioning the accuracy of the diagnosis or refusing to believe that their child had leukaemia. This reaction often represented a temporary coping mechanism as mothers attempted to process the emotional shock associated with the diagnosis.

*“I cannot accept the fact that my child was diagnosed with leukaemia due to his long, tiring treatment.” (*Mother of a 5-year-old male)

*“I never expected this and refused to believe my son was sick, thinking the tests were wrong.”* (Mother of a 5-year-old male)

*“I lost my mind. I didn’t believe them and called them liars.” (*Mother of a 4-year-old female)

#### Hindrances due to the political situation.

This theme reflects the structural and contextual barriers mothers encountered as a result of the political situation in the West Bank. Mothers described how movement restrictions, closures, checkpoints, and the broader context of conflict affected their ability to access essential healthcare services for their children with leukaemia. These conditions created additional stress and logistical difficulties, often delaying or complicating access to treatment. Furthermore, the political context contributed to social isolation during hospitalization, as family members were often unable to travel to provide emotional or practical support. This theme comprised two subthemes: limited healthcare access and feeling isolated due to closures.

**Limited healthcare access.** Mothers described significant difficulties in accessing healthcare services for their children due to mobility restrictions, checkpoints, and travel barriers between cities. These obstacles often increase the time and effort required to reach specialized treatment centres, adding further emotional and physical strain to an already challenging situation.

*“Treatment is available, but it’s easier to access in my city than here. The travel time and checkpoints cause me a lot of stress.”* (Mother of a 2-year-old female)

*“Difficulties reaching the hospital due to the war and road closures negatively impacted my mental state and my daughter’s condition.”* (Mother of a 3-year-old female)

Another challenge highlighted by participants was the distance between their homes and specialized treatment facilities. Many mothers reported that paediatric oncology services were not available in their districts, requiring them to travel long distances to receive appropriate care.

*“The services here are the worst due to the occupation; it’s very difficult to travel from city to city because of closures and checkpoints.”* (Mother of a 2-year-old female)

*“The distance has become a challenge for me in treating my child, especially with the war events the country is experiencing.” (*Mother of a 4-year-old female)

Some participants also emphasized the lack of specialized paediatric cancer services in nearby areas, which further increased their caregiving burden and emotional distress.

*“My district lacks leukaemia care facilities, so we must travel to another distant district for my child’s treatment.”* (Mother of a 5-year-old male)

*“I’m very far from the hospital, which makes me anxious about traveling because of the distance.”* (Mother of a 4-year-old female)

**Feeling isolated due to closures.** Another important challenge reported by mothers was social isolation during hospitalization, particularly when closures and checkpoints prevented family members from visiting or providing support. Many participants described feeling alone while caring for their hospitalized child for extended periods.

*“The situation became more challenging for me due to closures, as no one was able to come and help.”* (Mother of a 3-year-old female)

*“Being in the hospital for a long time was difficult for me because I was alone in another city.”* (Mother of a 3-year-old female)

*Several mothers explained that travel restrictions prevented relatives from reaching the hospital, leaving them without emotional or practical assistance during a highly stressful time.*

*“I often feel stressed from the closures, and when I come alone, I can’t see anyone.”* (Mother of a 2-year-old female)

*One mother also expressed fear and insecurity while staying in another city during a period of instability.*

*“I was afraid of being away from my children in another city because of the war and closures.”* (Mother of a 3-year-old female)

#### Impact of the child’s illness.

This theme reflects the profound impact of a child’s leukaemia diagnosis on family life, particularly on mothers who often assume the primary caregiving role. Participants described how the illness affected multiple dimensions of their lives, including emotional well-being, family relationships, financial stability, and daily routines. The diagnosis frequently required mothers to reorganize their priorities and responsibilities around the child’s treatment and care. As a result, many mothers experienced significant lifestyle changes and disruptions to family dynamics. This theme comprised seven subthemes: caregiver burden versus protective bonding, financial strain, loss of previous life, sibling neglect, shift in the role of the mother, marital life impact, and challenges in balancing home routine.

**Caregiver burden vs. protective bonding.** Mothers described experiencing a dual response to their child’s illness, characterized by caregiver burden alongside heightened protective bonding. On one hand, the intensive caregiving responsibilities created physical and emotional exhaustion. On the other hand, mothers often developed stronger emotional attachment and protective behaviors toward their child. Fear of infections, treatment side effects, and medical complications led many mothers to closely monitor their child’s activities and restrict social interactions.

*“I was afraid to take her to the park or let her play with other children, for fear that something would happen to her.”* (Mother of a 3-year-old female)

*“I imposed restrictions because I was concerned about my daughter’s health and immunity.”* (Mother of a 4-year-old female)

*“I was afraid to take her to the park or let her play with other children, for fear that something would happen to her.”* (Mother of a 6-year-old female)

**Financial strain.** Mothers also reported experiencing substantial financial challenges while caring for their children with leukaemia. Many participants had to leave their jobs or take extended unpaid leave to provide full-time care. At the same time, some families experienced reduced household income due to limited employment opportunities or movement restrictions. The combination of lost income and increased expenses created significant financial pressure for many families.

*“I left my job to care for my son as his illness worsened, since my husband and I were unable to work together.”* (Mother of a 12-year-old male)

*“Financially, the situation was difficult due to closures and lack of work.”* (Mother of a 3-year-old female)

*“Regardless of the main treatment, I needed money to cover all my and my child’s expenses.”* (Mother of a 6-year-old male)

**Loss of previous life.** Many mothers described a significant disruption to their previous lifestyle following their child’s diagnosis. Protecting the child from infections often requires limiting social interactions and reducing participation in social gatherings. As a result, mothers frequently withdrew from social activities and experienced increased isolation.

*“I lost interest in socializing due to fear that my daughter might catch something, as even a cold could harm her.”* (Mother of a 3-year-old female)

*“After the illness, I became more withdrawn and avoided visits and questions about my daughter’s condition.”* (Mother of a 3-year-old female)

Some mothers emphasized the contrast between their current situation and life before the diagnosis.

*“Before my daughter’s illness, life was truly free. There were no restrictions, and my daughter could play freely without limitations.”* (Mother of a 4-year-old male)

**Sibling neglect.** Another challenge reported by participants was the difficulty of balancing attention between the sick child and other siblings. Mothers explained that the intensive care needs of the child with leukaemia often required prioritizing that child’s health and treatment. Consequently, some mothers felt they were unable to devote sufficient attention to their other children.

*“My sick daughter takes priority over my other family responsibilities.”* (Mother of a 3-year-old female)

*“After my son was diagnosed, I feel that I have neglected the needs of my other children because all my attention is focused on him.”* (Mother of a 5-year-old male)

*“My middle daughter often complains and calls me an unfair mother, which shocks me.”* (Mother of a 4-year-old female)

Some mothers also described attempts to explain the situation to siblings in order to maintain family understanding.

*“I try to balance between them and explain to her that her brother is sick and needs special care because of his weak immunity.”* (Mother of a 10-month-old female child)

**Shift in the role of the mother.** Participants reported a significant shift in their family roles following their child’s diagnosis. Mothers often became fully dedicated to caring for the sick child, which required delegating other household responsibilities to family members.

*“I was greatly affected as a mother, neglecting my responsibilities toward my children, home, and husband, dedicating all my time to caring for my sick daughter.”* (Mother of a 3.5-year-old female)

*“I wasn’t doing anything while my family and my husband took care of everything, from studying to cooking.”* (Mother of a 5-year-old female)

**Marital life impact (positive and negative).** The illness also influenced marital relationships, producing both positive and negative effects. Some mothers described strengthened relationships with their spouses, characterized by emotional support and shared caregiving responsibilities.

*“The bonds with my husband have strengthened, and we support each other in tough times.”* (Mother of a 4-year-old male)

*“My husband provided great support, constantly reassuring me and taking care of everything, from getting supplies to looking after the other kids.”* (Mother of a 5-year-old male)

However, other participants reported that the emotional stress and logistical challenges associated with the illness created tension within the marital relationship.

*“At first, my husband and I distanced ourselves because of the sadness over our daughter.”* (Mother of a 3-year-old female)

*“I was anxious, irritable, and tense with my husband while we were away from home.”* (Mother of a 4-year-old female)

*“My marital life disappeared during this period, and our problems increased.”*(Mother of a 6-year-old male)

**Challenges in balancing home routine.** Finally, mothers described considerable difficulty in managing household routines alongside caregiving responsibilities. Frequent hospital visits, treatment schedules, and the physical and emotional demands of caring for a sick child often disrupted daily family routines.

*“I was extremely physically exhausted, sometimes sleeping without realizing it due to fatigue, yet I tried to balance everything.”* (Mother of a 4-year-and-10-month-old male)

*“I couldn’t balance everything; the physical and mental strain was overwhelming.”*(Mother of a 6-year-old female)

#### Resilience and endurance.

This theme reflects how mothers developed resilience while coping with the challenges associated with their child’s leukaemia diagnosis. Participants described several strategies that helped them endure the emotional and practical burdens of caregiving. These strategies involved drawing on external support systems, spiritual beliefs, and internal psychological strength. Together, these coping mechanisms enabled mothers to make sense of their experiences and maintain hope throughout their child’s illness trajectory. This theme comprised three subthemes: leveraging social support networks, fatalism and religious faith as a framework, and cultivating optimism and a positive outlook.

**Leveraging social support networks.** Mothers emphasized the crucial role of social suppor**t** in helping them cope with the demanding experience of caring for a child with leukaemia. Participants described receiving assistance from family members, particularly husbands and extended family, who provided both emotional and practical support. This support often included helping with household responsibilities and caring for other children, allowing mothers to remain present with their sick child during treatment.

*“There is nothing better than support; the people who stand by me in my times of need lighten my burden significantly.”*(Mother of a 4-year-old female)

*“The love and support from others eased my burden and helped me relax.”* (Mother of a 4-year-old male)

Many mothers reported that their families played an essential role in maintaining household stability while they focused on the sick child.

*“My husband’s family took care of my children and home in every aspect during my absence.”* (Mother of a 3-year-old male)

*“My family supported me emotionally and financially and helped with the house. Without them, I couldn’t have coped.”* (Mother of a 4-year-old male)

Husbands were frequently identified as key sources of support, sharing responsibilities and providing emotional reassurance during difficult times.

*“My husband was my biggest support in every aspect; both at home and in the hospital, he would work, take me to the hospital, encourage me, and fill the role of the family that wasn’t with me.”* (Mother of a 12-year-old male)

In addition, the emotional and informational support provided by healthcare professionals helped mothers feel reassured and more confident in managing their child’s treatment.

*“The medical team helped me immensely, both in treating my child and supporting my mental well-being; they were always there to answer my questions and assist me.”* (Mother of a 5-year-old male)

**Fatalism and religious faith as a framework.** Faith and spirituality emerged as important coping mechanisms for many mothers. Participants often described their child’s illness as part of God’s will or as a test of patience and strength, which helped them accept the situation and maintain emotional stability. Religious practices such as prayer, recitation of the Quran, and remembrance of God provided comfort and psychological relief during particularly stressful moments.

*“I turn to God for relief when overwhelmed and avoid thinking about the illness.”*(Mother of a 3-year-old female)

*“My son’s illness was a test from God for my family and me. Thank God, I am patient and content with God’s will and fate.”* (Mother of a 5-year-old female)

*“My coping mechanism involved repeating Surah Al-Baqarah, dhikr, prayers, and the patience God granted me.”* (Mother of a 4-year-old male)

**Cultivating optimism and a positive outlook.** Maintaining optimism and a positive outlook was another important strategy used by mothers to cope with the challenges of their child’s leukaemia diagnosis. Participants described consciously trying to remain strong and hopeful throughout the treatment process. Focusing on small improvements in their child’s condition helped mothers maintain motivation and emotional resilience.

*“I helped myself the most and tried to remain strong for my daughter.”* (Mother of a 3-year-old female)

*“Thank God, my daughter has improved a lot. Seeing her get better encourages me.”*(Mother of a 3-year-old female)

*“I decided I didn’t want to stay frustrated, so I started regaining my energy and taking care of the house on my own.” (*Mother of a 3-year-old female)

#### Mothers’ needs to enhance resilience.

This theme highlights the support systems and resources mothers identified as essential for fostering resilience while caring for a child with leukaemia. Participants emphasized that resilience depends not only on personal coping strategies but also on access to emotional, educational, and financial support. Three subthemes were identified: emotional and psychological support, educational and informational needs, and financial needs.

**Emotional and Psychological Support**. Mothers expressed a strong need for counselling, therapy, and structured psychological support to help manage the stress, anxiety, and grief associated with their child’s illness. Support interventions, such as individual counselling sessions, group therapy, and stress management programs, were seen as essential for maintaining emotional well-being. Recreational or relaxing activities were also considered important to allow mothers to recharge and sustain their caregiving role.

*“I need emotional and psychological support for ventilation.”* (Mother of a 6-year-old male)

*“We need psychological support from specialists who understand our fears and concerns.”*(Mother of a 4-year-old female)

*“The biggest need for a mother of a child with leukemia is psychological support and rest.”* (Mother of a 12-year-old male)

**Educational and informational needs.** Participants highlighted the importance of clear, accurate, and comprehensive information to effectively manage their child’s illness. Mothers described a need for guidance on the disease, treatment plans, potential side effects, and caregiving strategies. Educational support also included advice on medication management, nutrition, emotional care, and behavioral management during treatment. Additionally, mothers emphasized the importance of including siblings and other family members in educational programs to distribute caregiving responsibilities and reduce maternal stress.

*“I need counselling on how to care for my child.”* (Mother of a 4-year-old female)

*“Providing specialists to speak with us, especially for reserved mothers, can help explain the illness and alleviate future concerns.”* (Mother of a 3-year-old female)

*“Including the sick child’s siblings in educational programs about the illness would help reduce the mother’s burden.”* (Mother of a 5-year-old male)

*“I struggle to handle my daughter, so I need support on how to deal with her behaviour.”*(Mother of a 3-year-old female)

**Financial needs.** Financial support emerged as a critical requirement for mothers. Despite government health insurance covering treatment costs, families faced additional expenses such as transportation, accommodation, and lost income due to caregiving responsibilities. The economic burden added stress and could undermine mothers’ ability to focus fully on their child’s care. Participants emphasized that financial assistance would enhance their resilience and enable them to dedicate energy to caregiving without the constant pressure of economic constraints.

*“If there had been financial support alongside insurance and provided treatments, it would have helped a lot.”* (Mother of a 6-year-old male)

*“My biggest issues are worries about getting to the hospital, as I must take my child daily, and transportation is sometimes unavailable.”* (Mother of a 12-year-old female)

## Discussion

This qualitative study explored the experiences of mothers caring for children diagnosed with ALL in the West Bank, Palestine. The analysis identified several interconnected themes reflecting emotional, social, and structural challenges as well as resilience-building needs that shape mothers’ experiences. These themes include emotional issues, hindrances due to the political situation, the impact of the child’s illness, resilience and endurance, and mothers’ needs to enhance resilience.

Consistent with previous research, mothers experienced profound emotional distress at the time of diagnosis, including shock, disbelief, fear, anxiety, helplessness, and denial. These responses reflect the abrupt disruption of the mother’s expectations and the perceived threat to the child’s survival. Similar emotional trajectories have been widely documented across diverse cultural settings, particularly when children were previously perceived as healthy before diagnosis [16,17].

Beyond the initial diagnostic phase, mothers reported sustained psychological distress related to uncertainty surrounding treatment outcomes, fear of relapse, and concerns regarding long-term complications. This persistent anxiety has been described in the literature as a chronic condition accompanying parents throughout the cancer treatment trajectory [18–20]. Additionally, some mothers described early phases of denial, including questioning diagnostic accuracy or minimizing illness severity. Such reactions have been identified as common early coping mechanisms when parents are confronted with a life-threatening diagnosis in a child [21].

A central contribution of this study is the identification of political and structural barriers as important determinants of mothers’ caregiving experiences. Movement restrictions, military checkpoints, permit requirements, and road closures did not merely complicate access to healthcare; they interacted with the emotional, social, and practical demands of caring for a child with cancer. Uncertainty about travel, delays in reaching treatment, and difficulties navigating healthcare access added layer of stress to an already demanding caregiving situation. These constraints could intensify mothers’ feelings of fear, helplessness, and exhaustion, while simultaneously reducing the time and resources available to meet their children’s needs and maintain their own emotional well-being.

The consequences extended beyond healthcare access. Mothers described prolonged separation from family members during hospital stays, uncertainty during travel, and reduced opportunities to receive practical and emotional support from relatives and their wider social networks. Thus, structural barriers could contribute to a mutually reinforcing cycle in which difficulties accessing care increased emotional distress, while emotional and caregiving burdens further reduced mothers’ capacity to cope with prolonged uncertainty and treatment-related demands. In this context, coping resources may become particularly important, including the use of personal, familial, and spiritual resources where available.

These findings are consistent with previous evidence documenting systemic barriers to childhood cancer care in Palestine, including delayed referrals, permit denials, medication shortages, and limited diagnostic capacity within the West Bank [22,23]. A recent study in Gaza involving caregivers of children with cancer, including nine mothers, similarly identified substantial barriers to accessing diagnostic and treatment services, particularly within the referral process [24]. Evidence from other resource-constrained settings also indicates that psychological distress, financial strain, and disruption of family and social life are common experiences among caregivers of children with cancer [25,26], These findings suggest that psychological distress, financial strain, and social disruption are common features of childhood cancer caregiving.

However, the present findings highlight how these burdens may be intensified by conflict-related restrictions on movement, healthcare access, and continuity of treatment. Such structural constraints do not operate independently of mothers’ emotional and caregiving experiences; rather, they may increase uncertainty about the child’s care, prolong travel and hospital stays, and limit access to family and social support. Financial pressures associated with treatment and travel may further compound these challenges, reducing available coping resources and intensifying psychological distress, fatigue, and feelings of helplessness. This may create a mutually reinforcing cycle in which difficulties accessing care increase emotional and caregiving burdens, while heightened distress and limited support make it more difficult to manage the demands of prolonged treatment. Within this context, mothers may rely more heavily on available coping resources, including family and social networks and faith or spiritual beliefs.

The child’s illness profoundly disrupted family life, with mothers assuming primary responsibility for caregiving. Many adopted overprotective behaviors aimed at preventing infections and treatment-related complications, including restricting visitors and limiting social interactions. Similar patterns of heightened vigilance and maternal sacrifice have been reported among mothers caring for children with cancer in other settings [27].

Financial strain emerged as a significant challenge, particularly when mothers were compelled to leave employment to provide full-time care. This finding is consistent with evidence demonstrating the substantial economic burden associated with childhood cancer, including lost income, transportation costs, and indirect caregiving expenses [28]. In parallel, mothers reported a sense of loss related to their pre-diagnosis lives, characterized by social withdrawal, disrupted routines, and altered family dynamics. Comparable experiences of social isolation and lifestyle disruption have been widely documented among parents of children undergoing cancer treatment [29,30].

An additional important finding was the unintended neglect of siblings. Mothers described prioritizing the ill child’s needs, often at the expense of emotional and practical attention to other children, leading to feelings of guilt and psychological distress. Previous research similarly indicates that siblings may experience emotional neglect during prolonged treatment periods, while mothers struggle to balance competing caregiving demands [31–33].

Mothers also experienced substantial changes in their roles and daily functioning. Balancing intensive caregiving responsibilities with household duties, employment, and self-care was described as overwhelming, resulting in lives centered almost exclusively on the ill child. Comparable disruptions to mother roles and daily routines have been reported among mothers in Middle Eastern and other cultural contexts. Marital relationships were variably affected; while some mothers reported strengthened bonds and increased cooperation, others experienced tension and conflict due to chronic stress and emotional exhaustion. These mixed outcomes are consistent with evidence indicating that childhood cancer can both strain and strengthen spousal relationships [34].

Mothers employed a range of coping strategies, including reliance on family and social support, engagement with healthcare professionals, peer connections, and religious faith. Social support has consistently been shown to buffer psychological distress and promote resilience among mothers caring for children with cancer [35,36]. Peer support from other mothers of children with leukemia emerged as particularly valuable, providing shared understanding and emotional validation that could not be obtained through other sources [37]. Religious faith and spiritual practices constituted an important coping resource. Practices such as prayer, reading the Qur’an, and acceptance of divine will provide comfort and meaning during periods of uncertainty and distress. Similar reliance on spirituality has been widely documented in Middle Eastern and other religious societies, where faith plays a central role in coping with childhood illness and caregiving stress. Additionally, cultivating optimism and maintaining a positive outlook appeared as a key coping strategy among mothers facing their child’s leukemia diagnosis. Optimism emerged as a conscious effort to remain strong and hopeful and manage emotional distress during the prolonged treatment process. These findings are consistent with previous research showing that the parents’ optimistic attitudes toward the children’s cancer were the key factor that enhanced parental resilience [38,39].

While mothers in this study described a range of coping strategies, these behaviors also reflect broader needs to enhance resilience in the face of ongoing caregiving stress. Research shows that supportive care—including psychosocial counseling, peer support groups, and structured resilience-focused interventions—can significantly improve resilience and psychological well-being among parents of children with cancer. A systematic review and meta-analysis found that psychological interventions such as resilience training, self-disclosure, and peer support enhanced parental resilience with a large effect size, suggesting that structured support efforts can meaningfully bolster adaptive coping and mental health in caregiving parents [40].

Peer support and social networks serve as critical resilience resources, providing emotional validation and shared understanding not easily obtained through other sources; higher levels of social support are consistently associated with better coping and psychological outcomes in parents facing pediatric cancer [41]. Access to health information from professionals and supportive social relationships has also been shown to positively predict resilience, underlining the importance of caregiver education and social connectedness [42].

Interventions that address mothers’ spiritual, emotional, and practical needs—such as counseling sessions, peer support groups, resilience training workshops, education on caregiving challenges, and financial support—may not only reduce distress but also strengthen adaptive capacities that support long-term caregiving. Indeed, conceptual frameworks in pediatric oncology care highlight that resilience processes evolve and are shaped by coping strategies, social support, and interactions with care providers, pointing to multiple intervention points where health systems can support family well-being [43].

### Implications for practice and policy

The findings highlight several actionable opportunities to support mothers caring for children with leukaemia. First, integrating structured psychosocial support—including counselling, stress management interventions, and peer-support groups—into paediatric oncology care could reduce emotional distress and foster resilience. Second, providing educational resources about the child’s illness, treatment procedures, and home care management can empower mothers and improve caregiving confidence. Third, financial support mechanisms, such as transportation subsidies or caregiver stipends, may mitigate the economic burden that disproportionately affects mothers in conflict-affected settings. Finally, addressing structural barriers—such as permit delays and limited access to specialized care—requires coordination between healthcare providers, social services, and policy actors to improve timely and equitable care.

### Study strengths and limitations

This study employed a qualitative descriptive design that enabled an in-depth exploration of mothers’ experiences of caring for a child with leukaemia. The use of semi-structured interviews facilitated detailed accounts of participants’ emotional, social, and caregiving experiences, while qualitative content analysis provided a systematic approach to identifying and interpreting patterns within the data. A particular strength was the exploration of caregiving within the specific context of the southern West Bank, where healthcare access may be shaped by broader social and structural conditions. The findings therefore provide context-specific insights into how mothers perceive and navigate caregiving demands in this setting.

Several limitations should be considered when interpreting the findings. First, the study was conducted in a single paediatric haematology department. Consequently, the findings should not be considered generalizable to all mothers or families of children with ALL in Palestine or to families in other conflict-affected settings. Rather, the findings provide an in-depth account of the experiences of the participating mothers, and transferability to other contexts should be assessed in light of similarities in healthcare systems, sociocultural circumstances, and conflict-related conditions. Second, the study focused exclusively on mothers and did not include fathers, siblings, or other family members; therefore, the findings may not capture the full range of family experiences. Third, although participants described movement restrictions, checkpoints, permit requirements, travel uncertainty, and other structural challenges as influencing their caregiving experiences, these findings reflect participants’ perceptions within the study setting and should therefore be interpreted within this contextual scope.

### Recommendations for future research

Future research should examine caregiving experiences across multiple hospitals and geographic regions of Palestine and include larger and more diverse samples to explore similarities and differences across healthcare and social contexts. Studies involving fathers, siblings, and other family members could provide a more comprehensive understanding of the family-wide impact of childhood cancer. Longitudinal research could further examine changes in parental psychological distress, coping, and resilience throughout different stages of diagnosis and treatment. Future studies should also investigate how healthcare access, socioeconomic circumstances, and conflict-related structural conditions interact with caregiving demands. Intervention research is warranted to evaluate the effectiveness of psychosocial, educational, family-centred, and healthcare-navigation interventions in supporting caregivers and children in conflict-affected settings.

## Conclusion

This study provides an in-depth account of the emotional, social, and caregiving challenges experienced by mothers caring for children with leukaemia in the southern West Bank. Participants described psychological distress, caregiving demands, financial pressures, and difficulties associated with accessing healthcare, while drawing on social support, faith, and personal resilience as sources of coping. The findings suggest that mothers’ caregiving experiences are shaped not only by the demands of childhood cancer but also by the broader context in which care is delivered. However, given the single-hospital setting and purposive sample, these findings should be interpreted as context-specific rather than generalized to all families of children with ALL in Palestine or other conflict-affected settings. Supporting mothers and children may therefore require family-centred psychosocial care alongside efforts to strengthen continuity and accessibility of healthcare services. Further multi-site research is needed to determine the extent to which these experiences are shared across different Palestinian and conflict-affected healthcare contexts.

## Abbreviations

ALL: Acute Lymphoblastic Leukemia
